# Treating microsatellite unstable metastatic parathyroid carcinoma with anti-PD1 therapy

**DOI:** 10.1210/jcemcr/luag045

**Published:** 2026-03-24

**Authors:** Borna Amir-Kabirian, Radhwan AlRubaye, Benjamin Cadle, Daniel Mais, Zakaria Sibai, Rebecca Redman

**Affiliations:** Department of Medicine, Division of Medical Oncology and Hematology, University of Louisville, 529 S Jackston St, Louisville, KY 40202, USA; Department of Medicine, Division of Endocrinology, University of Louisville, 550 S Jackson St, Louisville, KY 40202, USA; University of Louisville School of Medicine, 323 E Chestnut Street, Louisville, KY 40202, USA; Department of Pathology, University of Louisville, #23 E Chestnut Street, Louisville, KY 40202, USA; Department of Medicine, Division of Endocrinology, University of Louisville, 550 S Jackson St, Louisville, KY 40202, USA; Department of Medicine, Division of Medical Oncology and Hematology, University of Louisville, 529 S Jackston St, Louisville, KY 40202, USA

**Keywords:** parathyroid carcinoma, microsatellite instability, immune checkpoint inhibitor

## Abstract

Parathyroid carcinoma (PC) is a rare endocrine malignancy that often clinically mimics primary hyperparathyroidism (PHPT) caused by parathyroid adenoma or hyperplasia. Complete resection of malignancy is the only curative treatment, and to date, there is no effective systemic therapy for advanced disease. A 60-year-old woman was referred for evaluation of hypercalcemia. Laboratory studies were consistent with PHPT. She underwent right superior parathyroidectomy, and pathology revealed PC with lymphovascular invasion. Postoperatively, calcium and parathyroid hormone levels normalized but recurred 6 months later. Imaging identified metastatic PC. Somatic genetic testing revealed a MutS homolog 2 (*MSH2*) splice-site mutation, indicating microsatellite instability. The patient started pembrolizumab immunotherapy, resulting in normalization of laboratory values and partial regression of metastases. This case highlights the diagnostic challenges of PC and underscores the potential role of immunotherapy in selected patients, particularly those with microsatellite instability, given the absence of established systemic treatment options.

## Introduction

Parathyroid carcinoma (PC) is a rare and aggressive endocrine malignancy, accounting for less than 1% of primary hyperparathyroidism (PHPT) cases [1, 2]. It typically presents in the fifth decade of life, with no sex predilection [1]. Most cases are sporadic, but familial forms occur in association with genetic syndromes such as hyperparathyroidism-jaw tumor syndrome (HPT-JT), multiple endocrine neoplasia type 1 (*MEN1*), and familial isolated hyperparathyroidism (FIHP) [1].

Clinically, PC is characterized by severe hypercalcemia above 14 mg/dL (>3.5 mmol/L) and up to a 10-fold increase in parathyroid hormone (PTH) levels [3]. Radiologically, it often appears as a large (>3 cm), heterogeneous mass with irregular borders and degenerative features, such as cystic cavities and calcifications [4]. Distinguishing it from benign parathyroid adenoma or hyperplasia remains challenging due to overlapping clinical, biochemical, and imaging features.

Definitive diagnosis is based on histopathologic examination demonstrating local tissue invasion. In many cases, metastasis is present at the time of diagnosis [5]. Although rare, PC is an aggressive malignancy with a poor prognosis, especially in advanced or metastatic cases, with 5- and 10-year survival rates ranging from 76% to 85% and 49% to 77%, respectively [3].

The mainstay of treatment is complete en bloc resection, though regional recurrence and distal metastasis occur in up to 60% of patients. Conventional chemotherapy and radiotherapy have shown minimal efficacy [6].

Microsatellite instability (MSI) is a hypermutator phenotype resulting from defects in the deoxyribonucleic acid (DNA) mismatch repair (MMR) system, leading to an increased mutation rate in tumor cells [7]. Identifying MSI in tumor cells is clinically important, as these tumors provide treatment options with immune checkpoint inhibitors [8]. Very few reports exist of PC with high MSI. In one study of 49 parathyroid adenomas, MSI was found to play little role in their pathogenesis [9]. We are presenting a case of microsatellite unstable metastatic PC to the lung with complete biochemical response and partial radiological response to programmed cell death protein 1 (PD-1) inhibition.

## Case presentation

A 60-year-old female was referred to endocrinology clinic for evaluation of asymptomatic hypercalcemia noted on labs for 3 years. She denied constipation, polyuria, muscle weakness, fatigue, or forgetfulness. She had no history of kidney stones, fragility fractures, or neck radiation. She had never been on lithium, thiazide diuretics, or any over-the-counter supplements. She reported no family history of calcium disorders or endocrine cancers. Neck examination did not reveal any palpable mass.

Laboratory tests revealed elevated calcium at 11.3 mg/dL (SI: 2.83 mmol/L) (reference range, 8.6-10.4 mg/dL [SI: 2.15-2.60 mmol/L]) and elevated PTH at 172 pg/mL (SI: 18.2 pmol/L) (reference range, 16-77 pg/mL [SI: 1.7-8.2 pmol/L]). The estimated glomerular filtration rate was 84 mL/min/1.73 m² (reference range, > 60 mL/min/1.73 m²), vitamin D 25-hydroxy was low at 14 ng/mL (SI: 35 mmol/L) (reference range, 30-100 ng/mL [SI: 75-250 nmol/L]), and the phosphorus level was normal. A 24-hour urinary calcium was within the normal limit at 150 mg/24 hours (SI: 3.75 mmol/24 hours) (reference range, 35-250 mg/24 hours [SI: 0.88-6.25 mmol/24 hours]). Kidney ultrasound revealed no kidney stones. Bone mineral densitometry study showed osteopenia in the lumbar spine and left forearm.

Based on biochemical and imaging findings, she was diagnosed with PHPT and referred to endocrine surgery for operative management. Preoperative neck ultrasound identified an oval-shaped, hypoechoic, and hypovascular lesion posterior to the right thyroid lobe measuring 1.0 × 1.5 × 1.2 cm.

She underwent a right superior parathyroidectomy. Intraoperatively, an enlarged parathyroid gland was identified, with its superior border involving the thyroid gland. Dissection was carried out to delineate the superior extent of the parathyroid, which was separated from the thyroid, requiring the removal of a small amount of thyroid tissue. Intraoperative PTH level decreased from 220 pg/mL (SI: 23.3 pmol/L) to 19 pg/mL (2.0 pmol/L) at 20 minutes. One week postoperatively, calcium and PTH levels normalized to 9.1 mg/dL (SI: 2.28 mmol/L) and 71 pg/mL (SI: 7.5 pmol/L), respectively.

**Figure 1:**
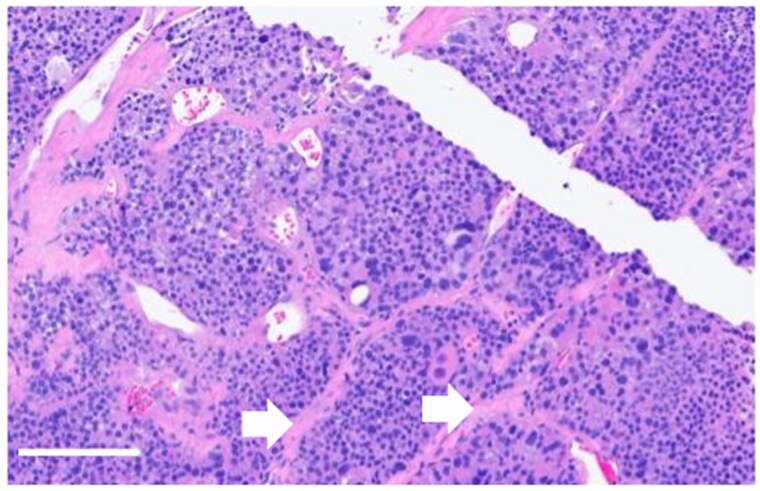
Hematoxylin and eosin-stained section showing sclerotic bands (white arrows) with significant nuclear atypia (scale bar = 100 μm, 20× magnification).

**Figure 2:**
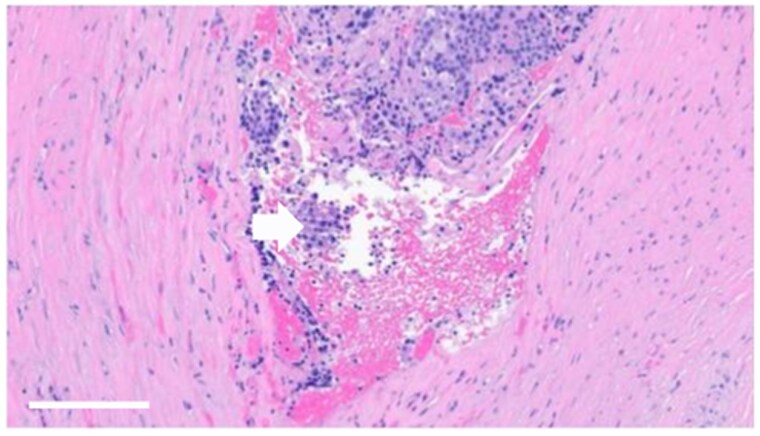
Hematoxylin and eosin-stained showing vascular invasion (white arrow) (scale bar = 100 μm, 20× magnification).

Histological examination revealed a 1.4 × 1.1 × 1.0 cm parathyroid neoplasm with characteristic endocrine cellular and nuclear atypia (Fig. 1). Immunohistochemistry was positive for PTH, confirming the diagnosis of PC. The malignant cells exhibited high nuclear grade and high mitotic index, consistent with high-grade malignancy. Malignant cells involved the cauterized superior edge of the resection, and there was focal necrosis with lymphovascular and capsular invasion. Strands of thick, fibrotic, capsule-like structures were seen, raising the suspicion of infiltration (Fig. 2).

Serum calcium and PTH remained stable for 4 months after surgery. A multidisciplinary tumor board reviewed the case, and a decision was made to proceed with a right hemithyroidectomy with a right-sided central neck lymph node dissection to reduce the risk of local recurrence due to margin involvement on initial pathology. Postoperative histopathology revealed no malignancy in the right thyroid gland or 6 examined lymph nodes.

## Diagnostic assessment

**Figure 3:**
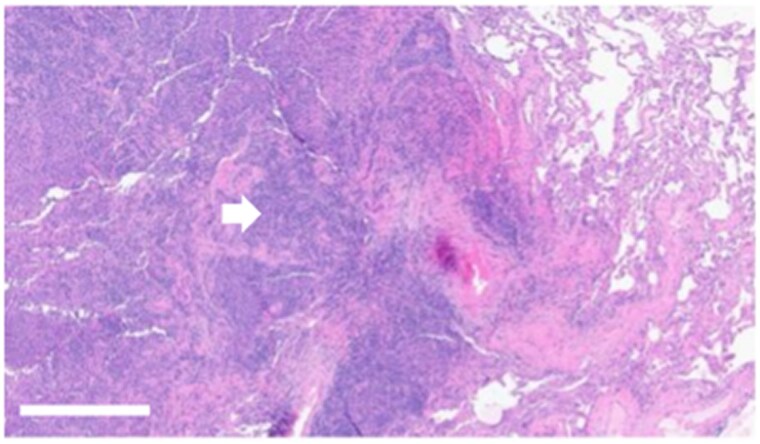
Hematoxylin and eosin-stained tumor displaying metastatic parathyroid carcinoma (white arrow) in the lung (scale bar = 100 μm, 10× magnification).

Six months postoperatively, laboratory studies showed recurrent hypercalcemia at 11.8 mg/dL (SI: 2.95 mmol/L) and elevated PTH of 264 pg/mL (SI: 28.0 pmol/L). Technetium-99 m sestamibi single-photon emission computed tomography/computed tomography (SPECT/CT) revealed a subcentimeter nodule posterior to the right lower thyroid lobe with no sestamibi uptake. Additionally, multiple bilateral pulmonary nodules were discovered, concerning for metastatic disease. Because of their size, they were not amenable to image-guided percutaneous sampling and required a left lower lobe wedge resection, which confirmed metastatic PC on pathology (Fig. 3). Somatic next-generation sequencing of this specimen demonstrated MutS homolog 2 (*MSH2*) splice-site alteration (c.645 + 1G > T) with a variant allele frequency (VAF) of 74.9%. This alteration was predicted to be pathogenic and indicative of MSI. Germline genetic testing was negative for *MSH2* mutation, cell division cycle 73 (*CDC73*), and *MEN1.*

The patient was referred to medical oncology to discuss systemic therapy. A CT scan of the chest revealed bilateral solid pulmonary nodules measuring 6 to 10 mm, without lymphadenopathy or mediastinal mass.

## Treatment

For hypercalcemia management, she received zoledronic acid 4 mg intravenously. Given the presence of bilateral lung lesions, the disease was considered unresectable. She was started on 200 mg of pembrolizumab, a humanized anti-PD-1 monoclonal antibody that was administered every 3 weeks.

## Outcome and follow-up

After 2 months of therapy, calcium level normalized to 9.3 mg/dL (SI: 2.33 mmol/L) and PTH normalized to 58 pg/mL (SI: 6.1 pmol/L), allowing discontinuation of the zoledronic acid. These levels remained stable at 8 months following the start of therapy with pembrolizumab.

CT scans 3 and 6 months after starting immunotherapy showed no evidence of local recurrence and a decrease in both the size and number of pulmonary nodules. Imaging findings were consistent with a partial response.

## Discussion

PC is a rare malignancy with a high rate of recurrence and distant metastases involving the lungs, liver, and bones. PCs are often difficult to distinguish from benign parathyroid adenoma or hyperplasia during initial evaluation for PHPT due to overlapping clinical symptoms, biochemical markers, and imaging features.

The World Health Organization has updated the classification criteria for PC, which now focus not only on histological features but also on evidence of invasive or metastatic disease. Another aspect of the update is the inclusion of molecular testing in the diagnostic framework [10].

The mainstay of treatment remains surgical excision with complete en bloc resection and ipsilateral thyroidectomy [6]. Approximately 40% to 60% of patients experience recurrence, typically within 2 to 5 years after initial resection. Recurrence is usually regional, involving cervical tissues or lymph nodes. Distant metastases occur in 25% of patients.

Data on systemic therapy for PCs are scarce. PCs have generally been deemed radio-insensitive, and chemotherapy has not been systemically evaluated in clinical trials [11]. Existing reports are largely anecdotal. Regimens such as dacarbazine, fluorouracil, and cyclophosphamide have been used with limited efficacy [12]. Given the absence of established treatment guidelines, targeted or immune-based therapies may represent viable options in selected cases.

The DNA MMR system is essential for correcting replication errors during DNA synthesis. When this system becomes deficient—known as deficient MMR (dMMR)—key proteins like MutL homolog 1 (*MLH1*), *MSH2*, MutS homolog 6 (*MSH6)*, and postmeiotic segregation increased 2 (*PMS2*) are often inactivated [13]. Tumors with dMMR cause mutations in the repetitive DNA sequences known as microsatellites, leading to high microsatellite instability (MSI-H). These accumulated errors create novel protein neoantigens that, when presented on the cell surface via major histocompatibility complex molecules, can stimulate an immune response.

dMMR tumors are characterized by dense immune cell infiltration, particularly of T lymphocytes expressing immune checkpoint proteins such as PD-1, cytotoxic T-lymphocyte-associated protein 4 (CTLA-4), and lymphocyte-activation gene 3 (LAG-3) [13]. This immunogenic tumor microenvironment creates tumors highly responsive to immune checkpoint inhibitors like pembrolizumab [13]. Clinical studies have demonstrated radiographic objective response rates up to 53% in patients with dMMR solid tumors treated with these agents [14].

In this patient, somatic next-generation sequencing demonstrated a pathogenic *MSH2* splice-site alteration (c.645 + 1G > T). This specific mutation disrupts the invariant guanine and thymine dinucleotide at a splice-site donor site, a position where even a single nucleotide substitution is considered pathogenic under established American College of Medical Genetics and Genomics and Association for Molecular Pathology genetic interpretation guidelines [15-17]. The VAF of 74.9% indicated nearly three-quarters of tumor cells carried this mutation. This elevated VAF suggested loss of heterozygosity, which supported Knudson’s 2-hit hypothesis, whereby tumor suppressor genes are inactivated through an initial mutation of one allele followed by loss of the remaining wild-type allele [18-20].

The most frequent genetic mutation identified in PC involves *CDC73*, and this has not yet led to the development of targeted therapies [21]. Somatic *CDC73* mutations can be detected in up to 80% of patients with sporadic PC, and approximately 30% of cases harbor germline *CDC73* mutations, suggesting that genetic predisposition plays an important role in disease pathogenesis [1, 3, 22]. Germline *CDC73* mutations are also known to be discovered in patients with HPT-JT and FIHP [1, 3, 22]. When *CDC73* genetic testing is negative, and the disease is present or recurrent and unresectable, next-generation sequencing could be considered for further mutation testing.

To date, only one case of MSI-high PC has been documented in the literature. In that case, a patient with biopsy-proven pulmonary metastases from PC was treated with 5 cycles of pembrolizumab, with normalization of calcium and PTH levels after 4 months and a 60% reduction in tumor burden on CT imaging [23]. Another case report published in 2024 described a case of locally recurrent, unresectable parathyroid cancer with a high tumor mutational burden (30 mut/Mb) treated with pembrolizumab plus external beam radiation, which resulted in normalization of calcium and PTH [22]. The clinical, molecular, and therapeutic characteristics of these cases, along with the present case, are summarized in Table 1.

**Table 1:** Comparison of clinical characteristics and treatment outcomes in 3 cases of parathyroid carcinoma treated with pembrolizumab

Characteristic	Park et al [22]	Katoh et al [23]	Current Case
Patient (age/sex)	65-year-old man with Lynch syndrome	49-year-old man with recurrent disease	60-year-old woman
Presentation/clinical features	Asymptomatic hypercalcemia; recurrent lung metastases	Recurrent unresectable PC; TMB-high	Asymptomatic hypercalcemia; recurrence with lung metastases
Serum calcium at presentation	Elevated	Elevated	Elevated
PTH at presentation	Elevated	Elevated	Elevated
Imaging/disease sites	Lung metastases	Unresectable locoregional recurrence and superior mediastinal mass	Locoregional and bilateral lung metastases
Tumor histopathology	PC; *MSH2* and *MSH6* deficient	PC; TMB-high	High-grade PC with invasion and necrosis
Genetic/molecular findings	MSI-high; germline MMR mutation (Lynch syndrome)	TMB-high; germline status NR	Somatic *MSH2* splice-site mutation; germline negative
Prior/concurrent therapies	Prior surgery	Multiple resections; RT combined with pembrolizumab	Parathyroidectomy; hemithyroidectomy; zoledronic acid
Biochemical response	Improvement in hypercalcemia	Improvement in hypercalcemia	Improvement in hypercalcemia
Pembrolizumab regimen	Pembrolizumab monotherapy 200 mg q3w	Pembrolizumab 200 mg q3w combined with RT	Pembrolizumab monotherapy q3w
Radiologic response	Approximately 60% tumor reduction	NR	Decrease in size and number of nodules (partial response)
Outcome	Durable partial response	Effective biochemical disease control during follow-up	Sustained biochemical control and radiologic response

Abbreviations: dMMR, deficient mismatch repair; MMR, mismatch repair; *MSH2*, MutS homolog 2; *MSH6*, MutS homolog 6; microsatellite instability; NR, not reported; PC, parathyroid carcinoma; PTH, parathyroid hormone; q3w, every 3 weeks; RT, radiation therapy; TMB, tumor mutational burden.

This case represents the third reported instance of pembrolizumab use as systemic therapy for recurrent and distant metastatic PC, demonstrating biochemical remission after 2 months and tumor regression on imaging after 3 months. This underscores the importance of assessing MSI status in PC, as it played a critical role in guiding effective therapy for our patient.

## Learning points

- PC can closely mimic benign adenomas or hyperplasia based on clinical presentation, biochemical findings, and imaging characteristics, making accurate diagnosis challenging.
- Molecular diagnostics, such as MMR and MSI testing, can guide the use of different therapies and influence treatment outcomes.
- Immunotherapy represents a promising treatment option in MSI high PCs, especially in advanced or recurrent PC where standard chemotherapy and radiotherapy may not be effective.

## Data Availability

Data sharing is not applicable to this article as no datasets were generated or analyzed during the current study.
